# Supplementary figures and images for: The Expanded Universe of Prokaryotic Argonaute Proteins
Source: mBio. 2018 Dec 18;9(6):e01935-18. doi: 10.1128/mBio.01935-18 (PMC6299218; doi:10.1128/mBio.01935-18)

pAgo\_genes ● 50 ● 100 ● 150 ● 200 ● 250

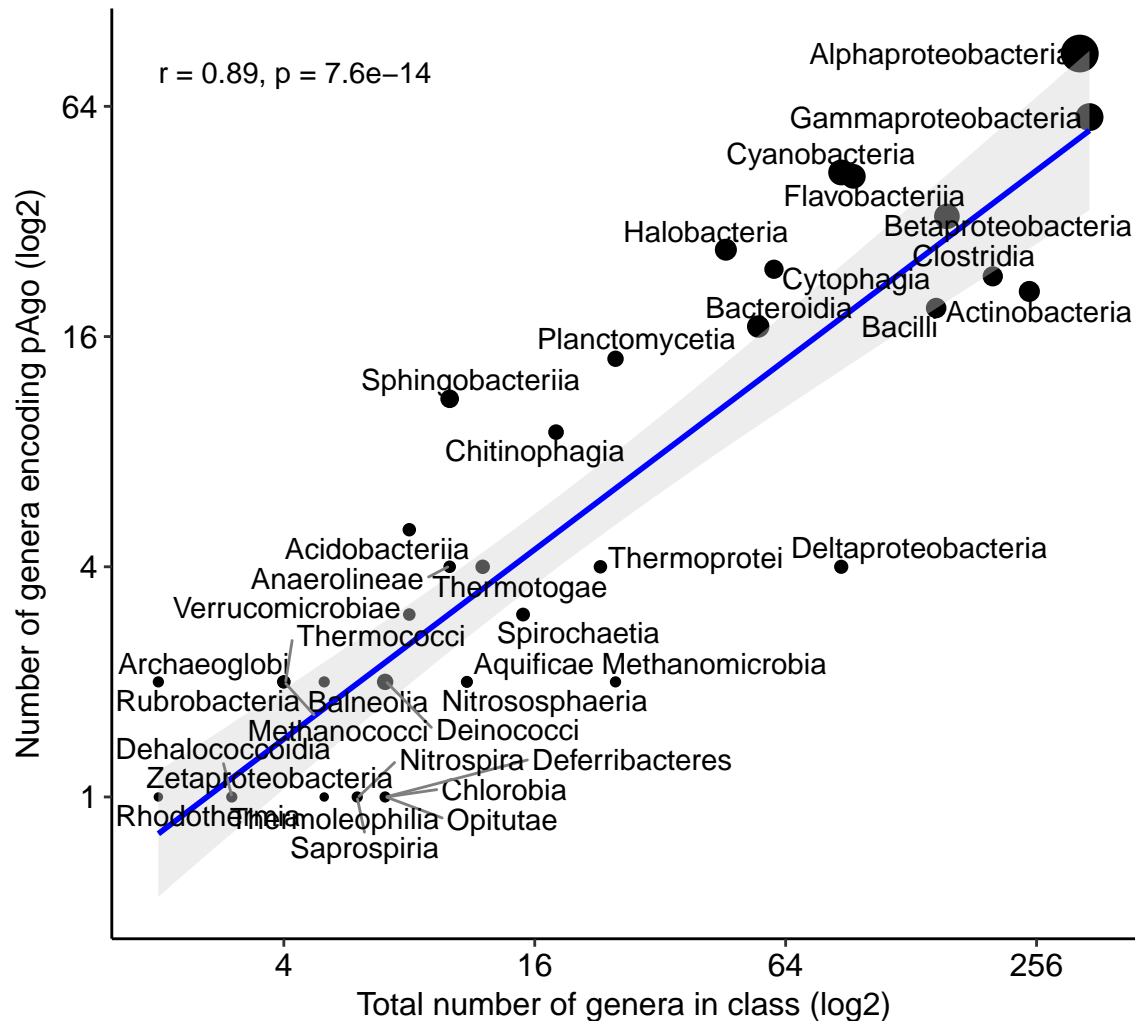

Supplement: FIG S1 [file mbo006184236sf1.pdf]

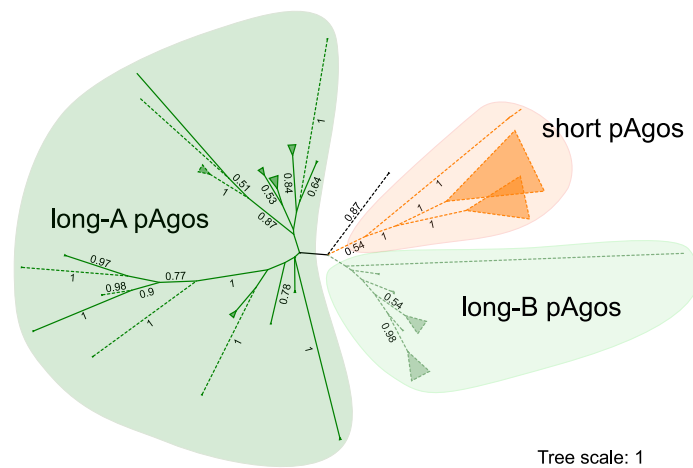

— PIWI domain with catalytic tetrad  
----- non-active PIWI\* domain

Supplement: FIG S2 [file mbo006184236sf2.pdf]

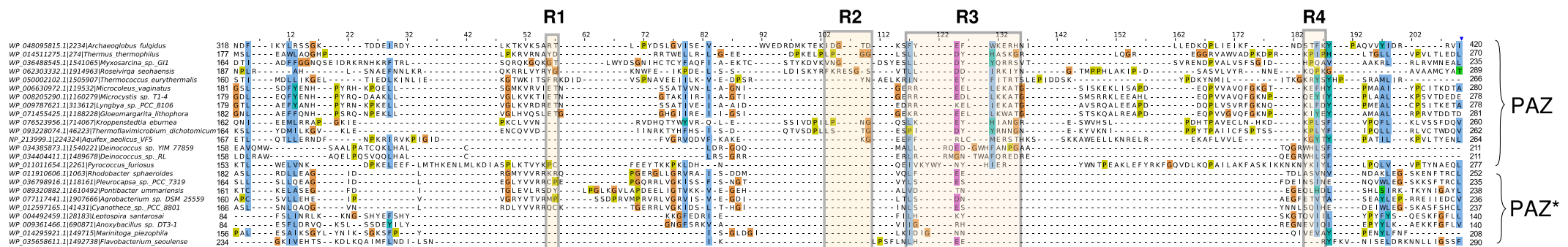

Supplement: FIG S3 [file mbo006184236sf3.pdf]
